# Contagious Period for Pandemic (H1N1) 2009

**DOI:** 10.3201/eid1605.091894

**Published:** 2010-05

**Authors:** Gaston De Serres, Isabelle Rouleau, Marie-Eve Hamelin, Caroline Quach, Danuta Skowronski, Louis Flamand, Nicole Boulianne, Yan Li, Julie Carbonneau, Anne-Marie Bourgault, Michel Couillard, Hugues Charest, Guy Boivin

**Affiliations:** Institut National de Santé Publique du Québec, Quebec City, Quebec, Canada (G. De Serres, I. Rouleau, N. Boulianne, A.-M. Bourgault, M. Couillard, H. Charest), Laval University, Quebec City (G. De Serres, M.-E. Hamelin, L. Flamand, J. Carbonneau, G. Boivin); McGill University–Montreal Children's Hospital, Montreal, Quebec, Canada (C. Quach); British Columbia Centers for Disease Control, Vancouver, British Columbia, Canada (D. Skowronski); Public Health Agency of Canada, Winnipeg, Manitoba, Canada (Y. Li)

**Keywords:** Influenza, contagiousness, shedding, pandemic, viruses, expedited, research

## Abstract

We estimated the proportion of persons with pandemic (H1N1) 2009 who were shedding infectious virus at diagnosis and on day 8 of illness. In households with confirmed cases, nasopharyngeal swabs were collected on all members and tested by PCR and virus culture. Of 47 cases confirmed by PCR at <7 days of illness, virus culture was positive in 92% (11/12) of febrile and 63% (22/35) of afebrile persons. Of 43 persons with PCR-confirmed pandemic (H1N1) 2009 from whom a second specimen was collected on day 8, 74% remained PCR positive and 19% were culture positive. If the 73 symptomatic household members without PCR-confirmed illness are assumed to have pandemic (H1N1) 2009, a minimum of 8% (6/73) of case-patients shed replicating virus on day 8. Self-isolation only until fever abates appears insufficient to limit transmission. Self-isolation for a week may be more effective, although some case-patients still would shed infectious virus.

## Abstract 2

Most infected persons shed live virus after fever abated.

Since April 2009, an influenza A virus, pandemic (H1N1) 2009, has spread to most countries of the world. Widespread susceptibility of persons <60 years of age may have facilitated rapid dissemination (*1*). Transmissibility of influenza viruses depends on duration of shedding, amount of virus shed, and other factors that may facilitate projection of virus into the environment, such as coughing or sneezing. Challenge studies in healthy volunteers inoculated with seasonal influenza viruses have shown that shedding generally coincides with symptom onset starting 1 day after inoculation, peaks on the second day, and generally ends 1 week after disease onset, on day 8 (*2*). Duration of shedding is greatly affected by age, and for seasonal influenza viruses is longer in young children than in adults (*3*–*5*). Since the emergence of pandemic (H1N1) 2009, the recommended duration of self-isolation has varied from complete resolution of symptoms to 1 day after fever has subsided (*6*,*7*). The objective of this study was to estimate the proportion of pandemic (H1N1) 2009–infected persons shedding infectious virus 1 week after illness onset.

## Methods

### Study Setting

**Figure 1:**
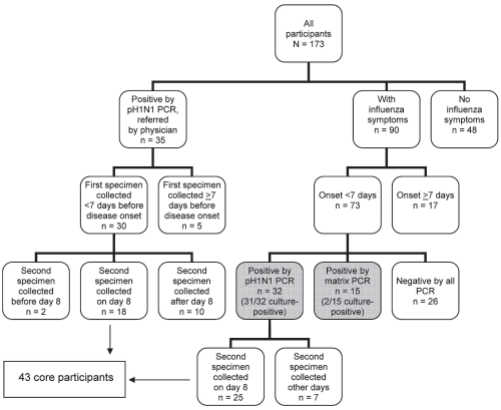
Flowchart of characteristics of 173 participants in study of shedding of pandemic (H1N1) 2009 virus, Quebec City, Quebec, Canada, May 27–July 10, 2009.

The research ethics committee of the Centre Hospitalier Universitaire de Québec approved the study. The prospective study was conducted during May 27–July 10, 2009, in Quebec City, Quebec, Canada. All participants were community based. Eligible persons were members of households in which at least 1 person with pandemic (H1N1) 2009 confirmed by reverse transcription–PCR (RT-PCR). Primary case-patients were either referred by their treating physicians or identified among community contacts of persons with laboratory-confirmed cases (Figure 1). At the initial home visit, a nurse obtained written informed consent from all household members and collected data for each household participant by using a standardized questionnaire. The questionnaire asked about basic sociodemographic characteristics (e.g., age, sex, occupation), presence of underlying medical conditions, presence of various symptoms or signs (i.e., fever, chills, cough, sore throat, rhinorrhea, arthralgia/myalgia, fatigue, dyspnea, headache, diarrhea, or vomiting), seasonal influenza vaccination history, and social and healthcare impact of their illness (i.e., missed workdays or schooldays, days spent in bed, medical consultations, emergency department visits, or hospital admissions).

### Nasopharyngeal Swabs and Laboratory Procedures

Nasopharyngeal (NP) secretions were collected from all household members, both symptomatic and asymptomatic, with NP swabs (Nylon Flocked Swabs; Copan Innovation, Brescia, Italy) that were inserted into 3 mL of universal transport medium (Copan Innovation). Patients with results positive by RT-PCR for pandemic (H1N1) 2009 before day 7 since disease onset had a second NP swab repeated on day 8. Persons still positive on day 8 were retested on day 11.

Nucleic acids were extracted from 200 µL of specimen by using the QIAGEN Viral RNA Mini Kit (QIAGEN, Mississauga, Ontario, Canada). Initially, pandemic (H1N1) 2009 was detected by using a conventional RT-PCR (pH1N1 PCR) specific for the hemagglutinin gene (*8*). RNA extracts from pH1N1 PCR–negative specimens were frozen at –80°C and, once specimen collection was completed, were retested by using a conventional RT-PCR assay for all influenza A viruses (matrix PCR), which has a higher sensitivity (*9*).

All samples with RT-PCR–positive results were cultured on MDCK cells in shell vials containing 1 mL of media to detect replicating viruses. Vials were observed daily for 7 days to detect cytopathic effects. Virus cultures were conducted on fresh specimens if they were positive by the pH1N1 PCR, whereas samples positive by the conventional matrix PCR had undergone 1 freeze-thaw cycle. All cultures showing cytopathic effects were sent to the Québec provincial reference laboratory for RT-PCR confirmation by using the previously described pH1N1 PCR.

### Statistical Analyses

We compared proportions and distributions using the χ^2^ test or Fisher exact test when appropriate. All statistical analyses were 2-tailed, and p values <0.05 were considered significant.

## Results

**Figure 2:**
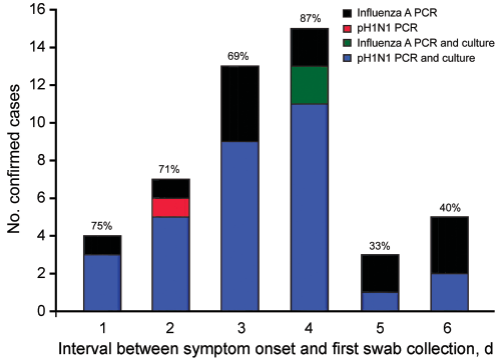
Positive results by PCR and culture for influenza A and pandemic (H1N1) 2009 virus (pH1N1) in 47 household contacts with laboratory-confirmed influenza, by delay between day of symptom onset and day of first swab collection, Quebec City, Quebec, Canada, May 27–July 10, 2009.

Of the 173 persons from 47 participating households, 35 with pH1N1 PCR–confirmed persons (index case-patients) were referred by their treating physicians. Among the 138 other participants (household or community contacts), 73 had respiratory symptoms for <7 days at the time of enrolment and 32 (44%) of 73 were positive by pH1N1 PCR (Figure 1, Table 1). Cell culture was also positive for 97% (31/32) of these pH1N1 PCR–confirmed cases. Of the 32 pH1N1 PCR–confirmed cases, 78% (25/32) had fever at some point since illness onset; at the time of specimen collection, 94% (30/32) had cough and 34% (11/32) were still febrile. Specimens were retested with a matrix PCR. All results from the pH1N1 PCR–positive participants were also positive by matrix PCR; of those who were negative by pH1N1 PCR, 37% (15/41) were positive. Of the 15 participants positive by matrix PCR, 13% (2/15) were positive by cell culture. Of the 47 confirmed cases (32 by pH1N1 PCR and 12 by matrix PCR), cell culture positivity varied from 69% to 87% for specimens obtained within 4 days after symptom onset and dropped to 33%–40% for specimens obtained days 5 and 6 after symptom onset (Figure 2).

**Table 1:** Characteristics of various subgroups of participants in assessment of length of shedding of pandemic (H1N1) 2009 virus, Quebec City, Quebec, Canada, May 27–July 10, 2009*

Characteristic	No. (%) core participants, n = 43	Symptomatic household or community contacts tested <7 days after symptom onset			
		Total no. (%), n = 73	No. (%) positive by pH1N1 and matrix PCRs, n = 32	No. (%) positive by matrix PCR only, n = 15	No. (%) negative by both PCRs, n = 26
Age group, y					
<10	20 (47)	24 (33)	13 (41)	4 (27)	7 (27)
10–17	11 (26)	15 (21)	9 (28)	1 (7)	5 (19)
>18	12 (28)	34 (47)	10 (31)	10 (67)	14 (54)
Sex					
F	24 (56)	41 (56)	17 (53)	8 (53)	16 (62)
M	19 (44)	32 (44)	15 (47)	7 (47)	10 (38)
Medical condition					
Any influenza-associated	8 (19)	15 (21)	6 (19)	4 (27)	5 (19)
Pulmonary disease	4 (9)	10 (14)	3 (9)	4 (27)	3 (12)
Influenza vaccination					
2008–2009 season	9 (21)	15 (21)	8 (25)	6 (40)	1 (4)
Clinical Illness†					
Fever	37 (86)	37 (51)	25 (78)	8 (53)	4 (15)
Cough	42 (98)	58 (79)	31 (97)	13 (87)	14 (54)
Sore throat	26 (60)	41 (56)	19 (59)	5 (33)	17 (65)
Myalgia or arthralgia	17 (40)	23 (32)	12 (38)	4 (27)	7 (27)
Fatigue	40 (93)	51 (70)	31 (97)	10 (67)	10 (38)
Headache	31 (72	45 (62)	24 (75)	9 (60)	12 (46)
Gastrointestinal symptoms	14 (33)	18 (25)	8 (25)	5 (33)	5 (19)
Fever and cough	36 (84)	35 (48)	24 (75)	8 (53)	3 (12)
Influenza-like illness	36 (84)	34 (47)	24 (75)	8 (53)	2 (8)

*pH1N1 PCR, PCR for pandemic (H1N1) 2009 virus. †Symptoms between onset of disease and first nasopharyngeal swab.

Of the 67 patients whose results were positive by pH1N1 PCR (35 case-patients initially referred by their physicians plus 32 detected among contacts), 62 were identified <7 days after symptom onset and 43 had a second swab collected on day 8 (Figure 1). Of these 43 cases (core participants), 47% were children <10 years of age, 26% were 10–17 years of age, and 28% were adults (>18 years of age) (Table 1). On day 8, 18 (42%) were still positive by pH1N1 PCR, and 14 others were positive only by matrix PCR, for a total of 74% (32/43) positive by any PCR method on day 8. (Table 2) Virus culture was positive for 19% (8/43) of the patients: culture was positive only for pH1N1 PCR–positive cases (8/18, 44%) but for none of the cases positive only by matrix PCR. PCR and virus culture positivity rates did not differ among age groups (Table 2). Only 5% (2/43) of case-patients were still febrile on day 8, but 91% (39/43) were still coughing. None of the 8 patients who had a positive virus culture on day 8 were febrile, but 7 (88%) were still coughing. Another swab was repeated on day 11 for 16 of the 18 case-patients who were positive by pH1N1 PCR on day 8 and 14 (88%) were still positive by at least 1 PCR (4 by pH1N1 PCR and 10 by matrix PCR). However, no specimen was positive by cell culture. On day 11, a total of 12 (86%) of the 14 PCR–positive case-patients were still coughing.

**Table 2:** PCR and virus culture positivity on days 8 and 11 of illness, by age group, Quebec City, Quebec, Canada, May 27–July 10, 2009

Time after illness onset	Age group, no. (%)			Total no. (%)	p value
	0–9 y	10–17 y	>18 y		
Day 8 of illness (2nd swab)	n = 20	n = 11	n = 12	n = 43	
pH1N1 PCR–positive specimen	10/20 (50)	3/11 (27)	5/12 (42)	18/43 (42)	0.43
Culture positive on pH1N1 PCR–positive specimen	5/10 (50)	1/3 (33)	2/5 (40)	8/18 (44)	1.00
Matrix PCR positive	5/10 (50)	6/8 (75)	3/7 (43)	14/25 (56)	0.53
Culture positive on matrix PCR–positive specimen	0/5	0/6	0/3	0/14	–
Any PCR positive	15/20 (75)	9/11 (82)	8/12 (67)	32/43 (74)	0.82
Culture positive on any PCR–positive specimen	5/15 (33)	1/9 (11)	2/8 (25)	8/32 (25)	0.51
Day 11 of illness (3rd swab)	n = 8	n = 3	n = 5	n = 16	
pH1N1 PCR–positive specimen	3/8 (38)	0/3	1/5 (20)	4/16 (25)	0.77
Culture positive on pH1N1 PCR–positive specimen	0/3	0/0	0/1	0/4	–
Matrix PCR–positive specimen	4/5 (80)	3/3 (100)	3/4 (75)	10/12 (83)	1.00
Culture positive on matrix PCR positive–specimen	0/4	0/3	0/3	0/10	–
Any PCR positive–specimen	7/8 (88)	3/3 (100)	4/5 (80)	14/16 (88)	1.00
Culture positive on any PCR positive–specimen	0/7	0/3	0/4	0/14	–

*pH1N1 PCR, PCR for pandemic (H1N1) 2009 virus.

Of the 73 symptomatic participants tested within 7 days after symptom onset, 15 of the 47 PCR–positive case-patients (in gray in Figure 1) were detected only by matrix PCR (2/15 cell culture positive) (Table 1). Of specimens positive by pH1N1 PCR, 97% (31/32) were culture positive at diagnosis, 44% (8/18) on day 8, and none on day 11 of illness. Of those whose results were positive by matrix PCR, 13% (2/15) were culture positive at diagnosis, and none were positive on days 8 or 11 of illness. Because virus culture was much less frequently positive for specimens positive only by matrix PCR (2/39, 5%) than for specimens positive by pH1N1 PCR (39/54, 72%), the 19% virus culture positivity on day 8 among core participants (all pH1N1 PCR positive) overestimated the true proportion positive at day 8. Assuming that none of the 15 case-patients with matrix PCR–positive results and 19% of the 32 case-patients with pH1N1 PCR–positive results would shed live virus on day 8, we can estimate that 6 [(19% × 32 pH1N1 PCR positive) + (0 × 15 matrix PCR positive)] of the 47 (13%) case-patients would still be positive by virus culture on day 8. If, instead, we assume that all 73 symptomatic participants were infected by pandemic (H1N1) 2009 and that positive cell culture on day 8 would be found only in patients positive by pH1N1 PCR, then 8% (6/73) still would be shedding live virus 1 week after illness onset. The real cell culture positivity rate on day 8 for all pandemic (H1N1) 2009–infected patients thus probably ranges from 8% to 13%.

## Discussion

Human challenge studies with seasonal influenza have shown that virus shedding after day 7 is rare (*2*), but clinical studies have shown that shedding may persist beyond that period in some populations, such as elderly persons, immunocompromised patients, and children (*3*–*5*,*10*–*12*). In a study among hospitalized persons infected with seasonal influenza A viruses, 54% remained positive by PCR beyond 7 days after symptom onset, and 29% were positive by cell culture (*13*). In another study, elderly hospitalized patients infected by influenza A (H3N2) viruses had higher virus loads than did outpatients, and their PCR positivity rate 1 week after disease onset was still high (57%) (*10*).

In this prospective study, the proportion of pandemic (H1N1) 2009–infected persons still shedding replicating virus on day 8 varied from 8% to 13%, with no difference between children and adults. None were still shedding infectious virus on day 11. With seasonal influenza, virus shedding may be longer in children because they have less preexisting immunity that would limit replication than in adults. However, children and adults <50 years of age appear equally susceptible to infection with pandemic (H1N1) 2009 virus, which would support our findings of comparable virus replication and shedding across age groups studied (*1*).

Our study had some limitations. First, our small sample size and study design may have limited our ability to directly measure culture positivity on day 8. Only a small number of patients had a specimen collected on day 8 and even fewer on day 11. In retrospect, a better design would have been to collect specimens from all 73 symptomatic household members on day 8, irrespective of the initial pH1N1 PCR result. That design would have enabled a more direct estimate of the proportion of patients who were culture positive on day 8, rather than the indirect approach we used. However, our extreme scenario (which assumes that all 73 symptomatic contacts were infected) provides the minimal positivity rate on day 8, and testing of all 73 on day 8 could only have found a proportion equal to or greater than our 8% estimate.

Second, our sampling methods could have influenced positivity rates. Although collection of NP specimens with a flocked swab is one of the best methods for obtaining specimens to detect influenza, those specimens might have been improperly collected by the nurses. Suboptimal collection of swabs would have yielded false-negative PCR or cell culture results, which in turn would have underestimated the proportion of patients shedding virus on day 8.

Third, PCR testing with the matrix PCR was conducted retrospectively on frozen specimens, and only 5% of those were positive by virus culture. A greater proportion of virus culture specimens might have been positive if those specimens had been processed immediately instead of going through a freeze-thaw cycle (*14*). Moreover, our study included only ambulatory patients ,whereas studies of seasonal influenza that include hospitalized or immunocompromised persons show prolonged shedding, contributing to the impression that our findings most likely underestimate the true proportion of case-patients still shedding virus on day 8. The strengths of our study include its prospective design in a family setting and its use of various methods, including 2 PCR assays and virus culture, to detect pandemic (H1N1) 2009.

Our results are consistent with other reports of virus shedding in pandemic (H1N1) 2009–infected patients. In Singapore, among 70 pandemic (H1N1) 2009–infected patients treated with oseltamivir and swabbed daily until virus clearance, 37% were PCR positive on day 7 of their illness and 9% on day 10 (*15*). No virus culture was performed in that study, so we cannot estimate the proportion of patients shedding infectious virus at these time points. However, even with oseltamivir treatment, the positivity rate by pH1N1 PCR on day 7 was similar to our own (42%) on day 8, and we can thus infer that the cell culture positivity rates also would be similar. In China, among 421 patients with serial swabs tested by real-time PCR but not cell culture, the median time from onset of disease to negative test result by real-time PCR was 6 days (range 1–17 days), indicating that 50% of patients were shedding virus >6 days (*16*).

A study conducted by Witkop et al. during a pandemic (H1N1) 2009 outbreak at the US Air Force Academy showed that 29% (31/106) of afebrile patients and 19% (11/58) of patients who had been symptom-free for 24 hours still shed viable pandemic (H1N1) 2009 virus. In their study, 24% of 29 swabs collected on day 7 and 13% of the 16 swabs collected on day 8 of illness were culture positive, despite the large proportion of patients prescribed antiviral drugs (*17*).

No definitive test is available for assessing the real contagiousness of a patient. The presence of replicating, and therefore infectious, influenza virus is an absolute prerequisite for contagiousness, but it does not necessarily imply it. Contagiousness depends on many factors, including viral load and presence of clinical characteristics contributing to spread of droplets (such as coughing, rhinorrea, or sneezing) and is affected by the number and proximity of contacts between a case-patient and a susceptible person. Nevertheless, our study raises concerns about current recommendations for self-isolation until only 24 hours after fever has subsided (*6*). With pandemic (H1N1) 2009, fever generally persists 1–4 days and may be absent in 6%–11% of patients (*1*,*15*). In our study, of the 32 pH1N1 PCR–positive household members who had been symptomatic for <7 days, 78% had fever at any time since onset of their illness, but only 34% were still febrile on the day they tested positive. Nonetheless, 97% of specimens obtained from these patients were positive by cell culture. Our sample size was insufficient to directly compare PCR or culture positivity by fever status or other symptom or severity indicator at specimen collection or as a component of the overall illness.

Before policy implications can directly follow from these findings, the association of self-isolation with substantial social impact needs to be carefully weighed against the possible benefits of reducing community transmission. In the general population, a 1-week self-isolation period seems more likely to prevent transmission than does isolation until fever has resolved. However, given that 8%–13% of patients may still shed infectious virus on day 8, longer periods of self-isolation for persons expected to come into contact with vulnerable persons (e.g., pregnant women, newborns, or immunocompromised persons) also may be prudent.
